# Moment-to-moment dynamics of ADHD behaviour

**DOI:** 10.1186/1744-9081-1-12

**Published:** 2005-08-01

**Authors:** Heidi Aase, Terje Sagvolden

**Affiliations:** 1 Norwegian Centre for the Studies of Conduct Problems and Innovative Practice, UNIRAND, University of Oslo, P.O.Box 1565 Vika, 0118 Oslo, Norway; 2 Department of Physiology, University of Oslo, Norway; 3 Centre for Advanced Studies (CAS) at the Norwegian Academy for Science and Letters, Oslo, Norway

**Keywords:** Reinforcement, response sequence, serial behaviour, variability, motor control, autocorrelations, behavioural units

## Abstract

**Background:**

The behaviour of children with Attention-Deficit / Hyperactivity Disorder is often described as highly variable, in addition to being hyperactive, impulsive and inattentive. One reason might be that they do not acquire complete and functional sequences of behaviour. The dynamic developmental theory of ADHD proposes that reinforcement and extinction processes are inefficient because of hypofunctioning dopamine systems, resulting in a narrower time window for associating antecedent stimuli and behaviour with its consequences. One effect of this may be that the learning of behavioural sequences is delayed, and that only short behavioural sequences are acquired in ADHD. The present study investigated acquisition of response sequences in the behaviour of children with ADHD.

**Methods:**

Fifteen boys with ADHD and thirteen boys without, all aged between 6–9 yr, completed a computerized task presented as a game with two squares on the screen. One square was associated with reinforcement. The task required responses by the computer mouse under reinforcement contingencies of variable interval schedules. Reinforcers were cartoon pictures and small trinkets. Measures related to response location (spatial dimension) and to response timing (temporal dimension) were analyzed by autocorrelations of consecutive responses across five lags. Acquired response sequences were defined as predictable responding shown by high explained variance.

**Results:**

Children with ADHD acquired shorter response sequences than comparison children on the measures related to response location. None of the groups showed any predictability in response timing. Response sequencing on the measure related to the discriminative stimulus was highly related to parent scores on a rating scale for ADHD symptoms.

**Conclusion:**

The findings suggest that children with ADHD have problems with learning long sequences of behaviour, particularly related to response location. Problems with learning long behavioural sequences may ultimately lead to deficient development of verbally governed behaviour and self control. The study represents a new approach to analyzing the moment-to-moment dynamics of behaviour, and provides support for the theory that reinforcement processes are altered in ADHD.

## Background

Attention-Deficit/Hyperactivity Disorder (ADHD) [1] is a behavioural disorder characterized by developmentally inappropriate levels of hyperactive, inattentive, impulsive, and variable behaviour. Impulsiveness is increasingly considered as a major behavioural symptom. A recent comprehensive theory of ADHD, the dynamic developmental theory (DDT), suggests two processes, altered reinforcement processes and inefficient extinction, as being causative of several of the behavioural symptoms in ADHD [2,3]. Specifically, the DDT suggests that delayed learning of complete and functional behavioural sequences may be causing the frequent shifts between activities, non-completion of tasks, lack of long-term planning, and deficient self-control that often are described as outcomes of impulsivity.

There is some support for the notion that ADHD behaviour may be characterized by hampered acquisition of complete and functional sequences of behaviour. First, children with ADHD did not perform sequences of arm movements as one functional unit, but were slower, showed greater variability in movement timing, and demonstrated longer inter-segment intervals than children without ADHD, who appeared to program the entire arm movements and executed the sequence as one functional unit that was temporally coordinated [4]. The children without ADHD in this study showed age adequate planned movement, while the children with ADHD resembled the performance of younger children using "on-line" or immediate-feedback monitoring [5]. Second, in a serial choice button-press task where advance information about the next steps in the sequence was gradually reduced, children with ADHD (and children with Tourette syndrome) showed increasing movement sequencing deficits compared to healthy controls as the level of advance information was reduced [6]. Third, on a task requiring high-level controlled processing (follow a target that randomly moves across the computer screen), preschool children at risk for ADHD were disproportionately more inaccurate and variable compared to healthy controls, children with borderline ADHD, and children with other psychopathology [7]. On a task requiring low-level processing (trace the mouse cursor within the limits of two lines), though, the difference between the groups was not significant. The authors concluded that deficits in self-control and self-regulation seemed to be present very early in the development of ADHD [7]. Finally, in a study investigating multitasking in ADHD and community controls, children with ADHD appeared to have a specific deficit in monitoring their ongoing behaviours and generating useful strategies for task completion [8].

### Reinforcement and behavioural sequences

The DDT suggests that dysfunctioning reinforcement and extinction processes can explain why symptomatic ADHD behaviour is acquired through dynamic interaction between the child and the environment throughout development [2,3]. Reinforcement and extinction are the main selection mechanisms of behaviour, and they are associated with dopaminergic activity [9]. According to the DDT, these mechanisms may operate constantly to reprogram neuronal connections by strengthening (reinforcing or potentiating) connections associated with reinforced behaviour, and at the same time weakening (extinguishing or depressing) other neuronal connections associated with nonreinforced behaviour [2].

**Figure 1:**
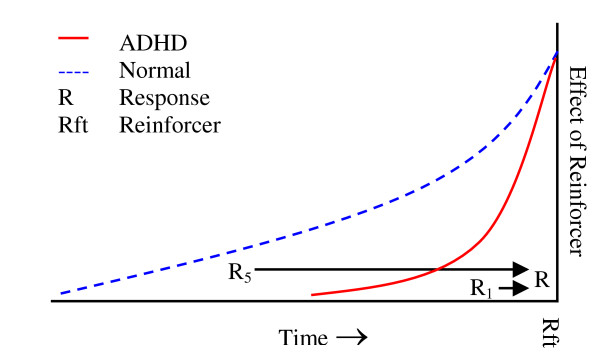
**Theoretical delay-of-reinforcement gradients. **The shorter and steeper delay gradient for ADHD (solid red line) implies that the relation between the response R_5 _and R will not be reinforced, while this relation will be reinforced with a normal delay gradient (broken blue line). The relation between R_1 _and R is close enough to be reinforced both when the delay gradient is short and when it is normal. A reinforcer will have almost the same effect on responses occurring immediately before reinforcer delivery with both a short and a long delay gradient, increasing the probability of repeating R with almost the same amount irrespective of the shape of the gradient.

On a behavioural level, reinforcers select responses by increasing the probability of repeating responses that produce reinforcers. Reinforcement as a process operates within a limited time window from the occurrence of the behaviour to the perception of the consequences of this behaviour. Altered reinforcement processes in ADHD may be described as a narrower time window than normal for associating behaviour with its consequences. A narrow time window may theoretically be depicted as a shorter and steeper delay-of-reinforcement gradient (called delay gradient from here onwards) (Figure 1). The delay gradient describes that the effect of a reinforcer is largest when it is delivered immediately after the response has been emitted, and wanes as a function of the delay in reinforcer delivery. The delay gradient thus depicts the relation between reinforcers and responses as an effect of time.

There may be many behavioural consequences of a shorter delay gradient in ADHD (see [2,3] for details); one of them being that it will only allow for short behavioural sequences to be associated with reinforcement. Thus, when there is a short time interval passing from the occurrence of a particular response to the presentation of a reinforcer, or a short sequence of responses is quickly and contingently followed by a reinforcer, this sequence will be strengthened by reinforcement with equal probability in ADHD behaviour and in normal behaviour (given that the delay gradients are at the same height at the time of reinforcement, see Figure 1). However, when reinforcers are delayed or follow after a long behavioural sequence, only the behaviour occurring within the restricted time window will be associated with the reinforcement and thus be strengthened. This may affect the establishment of serial ordering of behavioural units, which is fundamental to all forms of skilled action, from speech to typing to reaching and grasping [10,11].

Skilled performance involves hierarchically organized units of behaviour, where the higher levels are controlled by longer-term consequences, and lower levels are controlled by short-term consequences of individual movements [12]. The hierarchical organization of behavioural units seems to combine autonomous functions at low levels with the possibility of learning new operations at higher control levels: "If the 'vital' centers of the lowest levels were not strongly organized at birth, life would not be possible; if the centers on the highest levels ('mental centers') were not little organized and therefore very modifiable we could only with difficulty and imperfectly adjust ourselves to the circumstances and should make few acquirements" (Taylor, 1932 [13], p 437, cited in [12], p. 701). Hypothetically, a narrow time window for associating actions with its consequences may, throughout ontogenesis, detain the natural evolution of hierarchically controlled behavioural units of increasing complexity. Further, with a short delay gradient, a discriminative stimulus will not systematically be associated with reinforcement and the establishment of stimulus control will be slowed, resulting in impulsivity and increased behavioural variability [2]. This style of learning may ultimately impede the development of verbally governed behaviour and "self control", and will have consequences for how the child with ADHD understands and behaves within his or her environment.

### Purpose of the present study

The aims of the present study is to investigate the hypothesis that a short and steep delay gradient in ADHD will result in shorter and less predictable sequences in the behaviour of children with ADHD compared to controls, and to explore an untraditional way of investigating details in behavioural change. Traditional ways of analyzing behaviour in terms of means and standard deviations are too crude to identify moment-to-moment changes in behaviour, and may not reveal the behavioural dynamics underlying more global concepts like impulsivity and variability. The present study of behavioural sequences applies autocorrelations of consecutive responses as a means of studying moment-to-moment dynamics in responding. The data set was obtained from a study presented previously [14] and is presently analyzed in a different way.

The task was a computerized game where mouse clicks on one of two squares on the screen resulted in the presentation of a reinforcer. Reinforcers were delivered according to variable interval (VI) schedules, where responses result in a reinforcer after varying time intervals. With VI schedules a possible confusion of reinforcement effects with timing problems is avoided, as reinforcers are presented at unpredictable times [15]. All mouse clicks were recorded both in terms of where on the screen responses were placed (response location; spatial dimension) and response timing (temporal dimension). Thus, the present task allowed for analysis of both spatial and temporal aspects of behaviour.

## Methods

### Participants

The present study analyzed response data from 28 boys in the age range of 6:2–9:0 (yr:mo), 15 of whom had an ADHD diagnosis and 13 were healthy controls. These boys represented the young age group from the previous study. The older children (aged 9.5–12 yr) from that study were not included, as the comparison group showed to be inadequate (see [14], for discussion). The details of the recruitment and assessment procedures are presented elsewhere [14]; only an outline of group characteristics is provided here.

Participants in the ADHD group were referred from different clinical sources (school psychologists, child and adolescent psychiatric units, habilitation services, and a private specialist centre) and were included if they met the following criteria: 1) DSM-IV diagnosis of ADHD, of either three subcategories; 2) Full Scale IQ of at least 80; 3) no evidence of neurological disorder, psychosis, or pervasive developmental disorder; and 4) not taking any medication within the last 48 hours prior to testing. A diagnosis was confirmed after thorough clinical evaluation. Eight of the children had ADHD combined type, six had ADHD hyperactive / impulsive type, and one child had ADHD inattentive type. In addition, inclusion in the ADHD group warranted a score at or above the 95^th ^percentile on the Disruptive Behaviour Rating Scale (DBRS) [16] home or school version.

Comparison children were recruited from schools in urban and suburban areas of Oslo, the capital of Norway. Inclusion criteria were the same as for the ADHD group, except that no DSM-IV diagnosis should be present. In addition, they had to score below the sub-clinical range on the DBRS.

Intellectual ability was assessed by screening all children with four subtests (information, similarities, block design, and picture completion) of the WISC-R [17] (demographic variables outlined in Table 1).

**Table 1:** Means, Standard Deviations, and t-Tests for Age, IQ, and Questionnaire Scores

Groups					
Measure	**ADHD group ** N = 15		**Normal controls ** N = 13		*Group Comparison*^f^
		
	Mean	SD	Mean	SD	

**Age **yr:mo (SD in mo)	7:6	9,5	7:10	9,4	p > .282, n.s.
- range yr : mo	6:2 – 8:9		6:4 – 9:0		
**IQ **Full scale WISC-R	104.5^a^	10.5	114.5	12.5	**p < .04**
**DBRS**^c ^Teacher					
- inattention items	16.1	6.5	3.0	2.5	**p < .001**
- hyperactive/impulsive	18.4	8.0	2.0	2.5	**p < .001**
**DBRS**^c ^Parents					
- inattention items	16.7	6.0	4.1	2.1	**p < .001**
- hyperactive/impulsive	17.4	4.4	3.0	2.7	**p < .001**
**CBCL**^d^					
- externalised T-score	68.2	8.9	38.9	7.5	**p < .001**
- internalised T-score	57.2	9.5	40.0	5.7	**p < .001**
- attention factor T-score	63.8	9.9	51.1	2.1	**p < .001**
**TRF**^e^					
- externalised T-score	69.5^b^	10.9	46.1	6.8	**p < .001**
- internalised T-score	57.5^b^	9.5	45.0	7.2	**p < .001**
- attention factor T-score	60.5^b^	6.5	50.4	1.0	**p < .001**

^a^One case missing

^b^Two cases missing

^c^Disruptive Behaviour Rating Scale [16]

^d^Child Behaviour Checklist – parent form [30].

^e^Teacher Report Form [30].

^f^Two-tailed t-test for equality of means. Bonferroni adjustment of the p-level is set to p = .039. Results are highlighted after Bonferroni adjustment

### Procedure

The study was approved of by the Regional Medical Committee of Research Ethics. The parents of all the participants received written information about the study and gave written consent for their child to take part. All children were tested in quiet rooms, using the same tasks, apparatus, and test procedures (see [14] for details).

### Reinforcement Task

The task was designed as a computer game, and was presented to the child with the following instruction (translated from Norwegian): *"This is a game you may play now. It is a little strange, because I will not tell you how to play the game. Your task is to find out how the game works. You may use this mouse and move the arrow across the screen like this (experimenter demonstrates how to move the mouse). If you want to point, you can click with one of these buttons (experimenter points to the mouse buttons). You may talk while you are playing, but I will not answer any questions about the game. I will sit back here and write a little while you play. Do you understand your task? You may start now."*

The task was run on a Toshiba Pentium 300 CDT laptop connected to a colour monitor (see [14] for details). In brief, response squares were two same-sized, aligned squares on the screen, one in a light and the other in a dark shade of grey. The computer mouse was the response device. Clicks with either right or left button on one of the squares induced a brief change in the grey shade as feedback. Responses outside the squares were recorded, but did not result in any feedback. The dark grey square was the "correct" target. Clicks within this square would, with varying time intervals, result in a cartoon picture (reinforcer) appearing on the screen for 1.5 s together with a sound. Responses on the light grey square never resulted in cartoon presentations. Following reinforcer delivery, the squares switched sides at random, keeping the total number of presentations on each side the same.

Variable interval (VI) schedules of reinforcement, where responses may produce reinforcers after the passage of varying time intervals [15], were used. Two VI schedules alternated, each signalled by a separate screen background colour. The background colour functioned as the conditioned discriminative stimulus for the specific condition in operation, while the dark grey colour of the correct square was the discriminative stimulus for the reinforcer. The two schedules were a short VI (VI 2s) signalled by a navy blue background and a long VI (VI 20s) signalled by a bright yellow background.

There were two sessions, each of five segments. Each segment consisted of four short and four long intervals, and was terminated by a response and the delivery of a reinforcer. In the first session, the child would see a total of 40 reinforcers (cartoons). In the second session, the child received a small tangible reinforcer (trinket, coin or sweet) in addition to the cartoon picture. This was done in order to maintain reinforcer value. The entire task, including instruction, break between sessions, and a final, short interview with the child, took less than 30 min to complete.

### Data recording and statistics

Data were recorded by the laptop. Response side (left or right), response coordinates (i.e. the horizontal and vertical pixel that the tip of the arrow-shaped cursor touched when the child clicked a mouse button), and interresponse times (IRTs) were the recorded dependent measures. The individual IRT distributions were highly skewed with a long tail towards long IRTs. IRTs were therefore normalized by log transformations prior to analysis (logIRT = log10 (IRT/1000 + 0.001)).

#### Behavioural measures

Data from the VI 20s condition was used to study response sequences, as the short schedule only allowed for one or a few responses before a reinforcer was delivered. Predictability of responses over long sequences could theoretically be found according to different aspects of the behaviour, and in order to explore the different possibilities we computed three measures related to the spatial dimension and one measure related to timing. The first measure was a general *side response pattern*, i.e., whether consecutive responses were on the left or right side of the screen. Highly predictable responding would probably be related to the side where the correct target was positioned, and would thus be a complementary measure of stimulus control. Likewise, low predictability implies that responses are equally distributed on the two sides and is thus also a measure of low stimulus control. Next, due to the fact that reinforcers affect more responses than the one that produces it (Figure 1), predictable patterns in other aspects of response locations were explored. The *square response pattern *measure was based on the distance from the centre of the selected square, whether correct or not, to the spot where the response was placed. The *target response pattern *measure was based on the distance from the centre of the correct response target to where the response was placed. Both distance scores were in terms of pixels, with the centre of the square defined as 0,0. Finally, response sequences might be predicted by patterns in timing. Thus, *timing response patterns *were analyzed based on consecutive interresponse times (IRTs).

The ordering of responses spatially and temporally was assessed by autocorrelations. Autocorrelations (serial correlations) of each measure were correlations of consecutive values over five lags (correlations between n and n+1 response is the first lag, between n and n+2 response is the second lag, and so on up to correlations between n and n+5 response being the fifth lag). The autocorrelations were computed for each individual over sessions and segments. We predicted that the behaviour of children with ADHD would be characterized by lower autocorrelations and that their autocorrelation curves across lags would be steeper compared to the behaviour of healthy comparison children.

#### Statistics

Data were analyzed by means of SPSS 11.0 for Windows (SPSS) and Statistica 6.1 [18] program packages. The distance scores were computed as the square root of the sum of squared horizontal and vertical distances. Explained variance (autocorrelations squared) was analyzed using repeated measures ANOVA over sessions, segments, and lags. The ANOVA was supplemented with MANOVA. A multivariate approach to repeated measures of more than two levels is recommended because it bypasses the assumption of compound symmetry and sphericity [18]. Clinical group (2) was the between-group variable; and session (2), segment (5), and lag (5) were within-group variables.

#### Demographic data

Demographic and diagnostic measures of the ADHD and the comparison group were tested with two-tailed t-tests for equality of means and are displayed in Table 1. There were significant differences between groups on all measures including IQ, but not age. Whether to control for IQ difference has been debated (e.g., [19]) as undue weight may be put on the impact of IQ and remove variance that is a result of ADHD itself. Running the analyses with IQ as a covariate and without gave a similar overall picture of results. Thus, IQ was not included as a covariate in the reported analyses. (All non-published results may be obtained from the first author upon request.)

## Results

In general, acquisition of predictable response sequences was found in the spatial measures and not in the temporal measure. The ADHD group had significantly lower autocorrelations than the comparison group on two of the three spatial measures. Both groups had very low autocorrelations related to response timing. There was no Session effect on the three spatial measures, but the main effect of Lag was significant for all four measures. Results of the planned analyses with ANOVA and MANOVA are shown in Table 2.

**Table 2:** Results from repeated measures ANOVA and multivariate tests for repeated measures, of explained variance (squared autocorrelations)^1^

*Measure*	*Variable*	ANOVA		Multivariate	
		
		Df	F	Df	F
**Side Response Pattern**	Group (G)	1, 26	14,700***		
	Session (Ses)	1, 26	1,136	1, 26	1,136
	Segment (Seg)	4, 104	11,684***	4, 23	5,317**
	Lag	4, 104	143,735***	4, 23	42,369***
	G * Seg	4, 104	4,756***	4, 23	2,824*
	G * Seg * Lag	16, 416	1,810*	16, 11	1,675
					
**Square Response Pattern**	G	1, 26	10,981**		
	Ses	1, 26	2,505	1, 26	2,505
	Seg	4, 104	1,290	4, 23	0,721
	Lag	4, 104	91,581***	4, 23	28,576***
	G * Lag	4, 104	8,779***	4, 23	5,097**
	G * Ses * Lag	4, 104	2,812*	4,023	1,681
					
**Target Response Pattern**	G	1, 26	3,083		
	Ses	1, 26	1,061	1, 26	1,061
	Seg	4, 104	4,127**	4, 23	3,131*
	Lag	4, 104	201,232***	4, 23	60,921***
	G * Lag	4, 104	1,232	4, 23	3,172*
					
**Timing Response Pattern**	G	1, 26	0,155		
	Ses	1, 26	6,793**	1, 26	6,793**
	Seg	4, 104	2,125	4, 23	1,253
	Lag	4, 104	41,613***	4, 23	13,364***
	G * Ses	1, 26	5,116*	1, 26	5,116*
	G * Ses * Lag	4, 104	4,326**	4, 23	2,499

* : p < .05; **: p < .01; ***: p < .001

^1^Due to a very large number of possible interactions, only main effects and significant interactions involving the group variable are reported. All non-published results may be obtained from the first author.

### Side response pattern

**Figure 2:**
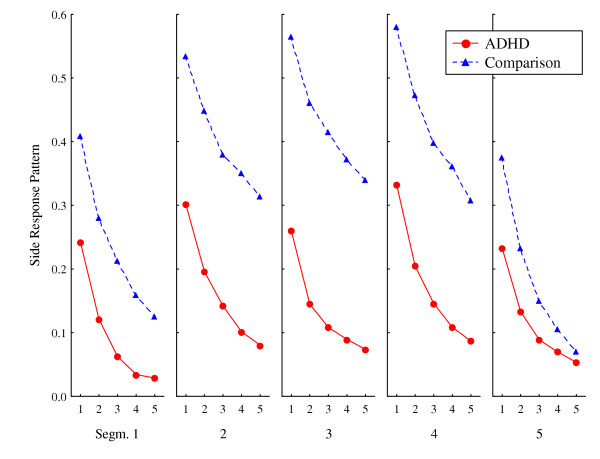
**Response pattern according to side of the screen. **Predictability of which side of the screen consecutive responses were placed, depicted as mean explained variance (autocorrelations squared), by segments (1–5) and lags (1–5 per segment), for ADHD and comparison groups. Graphs show means of session 1 and session 2.

This measure assessed whether responding predictably would continue on the same side or vary unpredictably between sides, irrespective of on which side the correct response target was displayed (see Behavioural measures in Methods for a detailed description of the variables). Highly predictable responding over lags would imply that behaviour was ordered in sequences related to side. Less variance was accounted for in the ADHD group than in the comparison group. For the ADHD group, explained variance in the first lag across segments was low (range 0.33 > mean R^2 ^> 0.22, median R^2 ^= 0.26), while it was in the upper range for the comparison group (0.62 > mean R^2 ^> 0.35; median R^2 ^= 0.50) (Figure 2). Actually, in some segments, explained variance over all five lags was higher for the comparison group than in the first lag for the ADHD group. In addition, explained variance in these segments did not descend much from the first to the fifth lag (not shown), indicating highly predictable response sequences for up to six responses for the comparison group.

There were significant main effects of Group, Segment, and Lag (Table 2). In addition, there was a significant two-way interaction between Group and Segment. In the comparison group there was a general within-session upward trend from segment 1 to 4, indicating a learning effect, but this trend did not continue into the last segment of each session (see Figure 2). The ADHD group did not show a similar pattern; explained variance did not improve during the task. This group difference was supported by a significant three-way ANOVA interaction between Group, Segment, and Lag. However, this interaction was not confirmed by the MANOVA.

### Square response pattern

**Figure 3:**
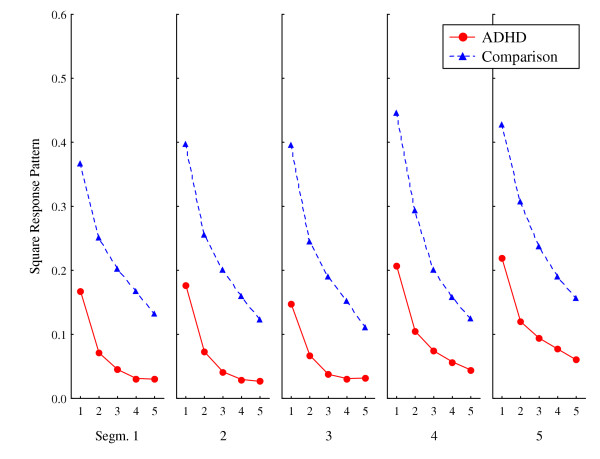
**Response pattern according to distance from the centre of a square. **Predictability of distance from the centre of the chosen square, whether correct or not, to where on the screen consecutive responses were placed. Curves show mean explained variance (autocorrelations squared) by segments (1–5) and lags (1–5 per segment), for ADHD and comparison groups. Graphs show means of session 1 and session 2.

This measure assessed to what degree the children tended to respond in any predictable pattern in terms of the distance between responses, anchored to the centre of the square that responses were placed within (whether it was the correct response target or not). Highly predictable responding would imply that behaviour was ordered in sequences of similar distances between responses. Again, the explained variance for the ADHD group was lower than for the comparison group (Figure 3). Explained variance in the first lag for the ADHD group were in the low range (0.25 > mean R^2 ^> 0.13; median R^2 ^= 0.18), indicating that there was rather low predictability from one response to the next. For the comparison group, explained variance in the first lag was higher (0.51 > mean R^2 ^> 0.22; median R^2 ^= 0.42).

There were significant main effects of Group and Lag, but not of Session and Segment (Table 2). A significant ANOVA interaction between Group and Lag was confirmed by the multivariate analysis, while the significant interaction between Group, Session, and Lag was not confirmed by the MANOVA.

### Target response pattern

**Figure 4:**
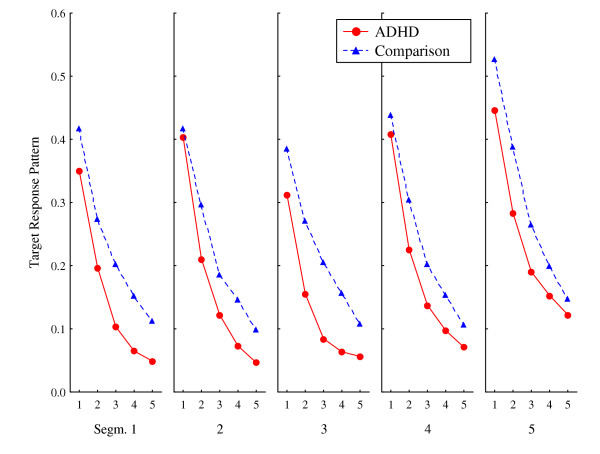
**Response pattern according to distance from the centre of the correct target. **Predictability of distance from the centre of the correct target to where on the screen consecutive responses were placed. Curves show mean explained variance (autocorrelations squared) by segments (1–5) and lags (1–5 per segment), for ADHD and comparison groups. Graphs show means of session 1 and session 2.

This measure assessed patterns of response placements in terms of distance from the centre of the correct square. Highly predictable responding would imply that behaviour was ordered in sequences of similar distances between responses, specifically related to the centre of the correct square. Again, low explained variance indicated high variability in responding. Explained variance in the first lag across segments of the ADHD group was in the middle range (0.46 > mean R^2 ^> 0.27; median R^2 ^= 0.40), and slightly higher for the comparison group (0.60 > mean R^2 ^> 0.36; median R^2 ^= 0.41) (Figure 4).

The main effects of Segment and Lag were statistically significant, but not the main effects of Group or of Session (Table 2). The MANOVA, but not the ANOVA, showed a two-way interaction between Group and Lag. There were no other significant interactions. As can be seen in Figure 4, the curves are quite similar for the two groups, but explained variance tends to be lower in lags 2–5 in the ADHD group compared to the comparison group.

### Timing response pattern

The development of patterns in response timing was investigated by means of consecutive interresponse times (IRTs). Explained variance of the first lag was generally very low and not significantly different between the groups (0.06 > mean R^2 ^> 0.12; median R^2 ^= 0.06 for ADHD, and 0.16 > mean R^2 ^> 0.02; median R^2 ^= 0.1 for comparisons). The significant main effect of Session showed that explained variance was lower in the second session than in the first, particularly for the comparison group. The main effect of Lag was significant, while the main effect of Segment was not. There was a significant interaction effect between Group and Session, showing that while the ADHD group had lower explained variance than the comparison group in the first session, the comparison group had lower explained variance than the ADHD group in the second session. The significant interaction between Group, Session, and Lag in the ANOVA was not confirmed by the MANOVA.

### Relation to clinical scores

**Figure 5:**
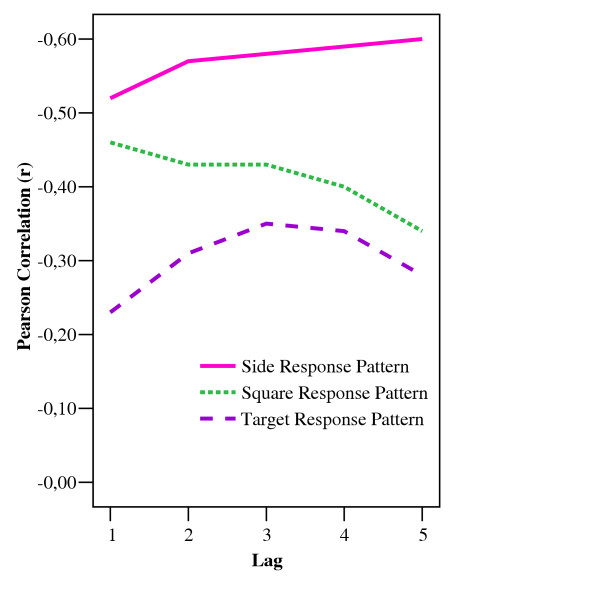
**Relation between explained variance of responding over lags and clinical scores. **A correlogram showing the relation between mean explained variance (autocorrelations squared) by lags and scores on the hyperactive / impulsive items of the Parent form of the Disruptive Behaviour Rating Scale (DBRS) for the three spatial measures in session 2. The relation was negative; i.e. high scores on the DBRS predicted low scores on the autocorrelations.

The dynamic developmental theory (DDT) argues that a shortened delay gradient probably relates more to the hyperactive / impulsive and the combined subtypes of ADHD than to the inattentive subtype [2]. The present sample did not allow for a differential analysis of clinical subtypes. However, the relation between the individual scores on the sub-dimensions of ADHD and explained variance on the spatial measures could be computed and would indicate if either the hyperactive / impulsive dimension or the inattentive dimension were better predictors of the explained variance. Thus, mean explained variance across lags for each spatial measure was correlated with sum scores of the inattentive and the hyperactive / impulsive dimensions on the DBRS parent form [16]. The correlations showed an inverse relationship between scores on the rating scale and predictability of responses, indicated by negative values (Figure 5). Correlations between the explained variance and the two sub-dimensions on the DBRS were not very different, but explained variance from session 2 was better than session 1 as a predictor of scores on the DBRS. The explained variance of the *side response pattern *was the best predictor of scores on the DBRS, with increasing correlation over lags (Figure 5, solid line).

## Discussion

The present study investigated the predictability of behavioural sequences in ADHD and in comparisons. The aims of the study was both to investigate the hypothesis that a shortened delay gradient in ADHD would result in short and less predictable response sequences in the behaviour of children with ADHD [2], and to explore the use of autocorrelations as a way of analyzing details in behavioural dynamics. Consecutive responding was studied in terms of three spatial and one temporal response dimensions. The results showed that predictable response sequences did develop according to the spatial response dimensions, but not according to the temporal dimension. Importantly, on the spatial dimensions, predictability of response sequences was considerably lower for the ADHD group than for the comparison group, as shown by significant interactions involving group and lag (number of consecutive responses). In addition, the overall explained variance of the ADHD group responding was significantly lower than that of the comparison group according to *side response pattern *and *square response pattern*, but not according to *target response pattern *or *timing response pattern*. This was supported by the high correlations between variance accounted for in consecutive response lags of these two spatial measures and the scores on both hyperactive/impulsive and inattentive clinical dimensions (Figure 5). Thus, the disorganized behaviour observed in the ADHD group may be a general behavioural feature captured by the clinical scoring by teachers and parents.

The *side response pattern *assessed whether responding could be predicted according to side (left or right) of the screen, irrespective of on which side the correct target was displayed. The significant group difference implied a highly predictable pattern in the comparison group, indicating that these children varied their responding between sides almost only to the extent that the correct target square switched sides on the screen. This was supported by their mean percent correct responding at 87% during stable state [14], demonstrating good discriminative control. The ADHD group, however, varied their responding between sides even though the correct target square was still on the same side, and they never exceeded 61% correct responses [14]. The comparison group showed predictable sequences of up to six responses where variance accounted for was larger for the n+5 response than for n+1 response in the ADHD group (e.g. Segments 2 and 3 in Figure 2).

However, there was a drop in explained variance in the comparison group at the end of each session (Figure 2, segment 5). This may have been an effect of the schedule, as the VI schedule was made up of predefined interval lengths, with more of the short intervals in the beginning of the session and more of the longer intervals towards the end. Hence, the disappearance of regular, predictable responding seen in the comparison group may have been the result of inter-reinforcement intervals being very long. This effect was not seen in the ADHD group.

The *square response pattern *and the *target response pattern *were both computed in order to explore possible patterns related to the spatial distance between responses, anchored to the centre of the squares. Organizing responding within the squares was not differentially reinforced. However, a shortened time window available for strengthening connections between events, as suggested by the DDT [2], predicts less systematic response patterns in ADHD compared to normal, which was found. The *square response pattern *measured distances from the centre of the chosen square to where the response was placed. The comparison group showed significantly more predictable responding than the ADHD group, both overall and across lags, indicated by the significant interaction between group and lag. The *target response pattern *measured the distance from the centre of the correct square to where the response was placed. The similar magnitude of explained variance (about 40–50%) in the first lag of the two groups indicates high predictability from response n to n+1 when in the correct square, while the ADHD group varied more on consecutive responses as indicated by the significant interaction between group and lag.

Increased variability is a consequence of reduced stimulus control. This is seen in the ADHD group in terms of the *side response pattern*. Therefore, it might be argued that the reduced predictability found in both *square response pattern *and *target response pattern *is a consequence of larger arm movements in the ADHD group because of more varied responding from side to side, rather than an effect of inefficient reinforcement of response placement within a square. The present analysis did not allow identifying predictability of response placements when consecutive responding was within the same square, which involves smaller movements. However, disentangling the variability related to larger arm movements and the variability related to decreased stimulus control (revealed as more varied responding) may not be feasible. Further, it may be speculated that, since the striatum, which is involved in the planning and execution of motor actions, and the nucleus accumbens, which is involved in learning and reinforcement, both receive important dopaminergic afferents, these functional processes may both be impaired in an individual with ADHD [2].

Acquisition of functional behavioural sequences may be related to processes involved in habit learning. Habit learning is characterized by a transition from response-consequence associations that are flexible and sensitive to reinforcement devaluation, to stimulus-response associations that are less flexible and sensitive (e.g. [20]). Thus, the initial part of habit learning may mainly involve activity in the mesolimbic dopaminergic branch, while the established habit and the skilled execution of the motor sequence may mainly involve the nigrostriatal dopaminergic branch. It may be argued that the present study is mainly concerned with acquisition, since the task was new and relatively short. On the other hand, animal model studies have indicated that operant learning rapidly become habitual when the contingency between the response and reinforcer is weakened by using interval schedules [20], as used in the present study. Hence, it might be speculated that the control group rapidly developed a habit, while this process was hampered in the ADHD group. Whether the present findings are due to impairments in habit formation or motor control related to the striatum, or to learning deficits related to nucleus accumbens cannot be settled, but the DDT predicts dysfunction of both processes [2].

Explained variance of first lag (and following lags) was too low to conclude that there was any predictability in IRTs. Thus, there was no *timing response pattern*. Speculatively, this lack of a predictable timing pattern could be related to the visuo-spatial nature of the task, both in terms of response alternatives (*where*, not when) and in terms of the reinforcer. There is some evidence that striatal neurons involved in sequential habit learning may encode visuo-spatial information rather than temporal [21].

The present findings suggest that the learning of coherent and predictable behavioural sequences will be difficult in children with ADHD, probably because the time window available for reinforcers to work is narrower in ADHD compared to normal. Skilled performance is characterized by responses emitted with brief interresponse intervals, smooth transitions between responses, and efficient coordination of consecutive movements so that a whole sequence is conducted in a predictable manner (e.g. [22]). Reinforcers are actively involved in the selection and shaping of responses, in chunking discrete responses into larger behavioural units, and in establishing relations between antecedent stimuli and behavioural units into sequences that together constitute a complete action (e.g. sequences of behavioural units comprising the entire action of typing a word, writing your signature, or playing an arpeggio on the piano). With a shorter delay gradient, the whole process of organizing hierarchical structures of actions comprised of functional units of behaviour may be hampered in ADHD. Although speculative, the present results indicate a different style of learning in ADHD, probably brought about by inefficient dopaminergic processes, which might be regarded as a separate endophenotype of ADHD [3,23] that forms the development of behavioural characteristics of variability, impulsiveness, lack of goal-directed behaviour, and hampered development of self-control. Such a learning style may explain the heterogeneity in symptom presentations among individuals with ADHD, because the behaviour of different individuals will be the result of interactions with different environmental contingencies.

Other interpretations are possible, however. It has been suggested that the length of delay gradients may be dependent on working memory (WM) capacity because the ability to relate sensory information to responses and reinforcing stimuli seems to correlate with ongoing neuronal activity in prefrontal cortex [24]. Behavioural sequences or serial movement has been related to WM capacity, as it has been argued that serial sensory information is stored in WM and converted into a movement program with the help of visual stimuli [25]. There is obviously some kind of memory process involved in reinforcement, and the delay gradient may as well be described as the result of pairing the reinforcer with the fading of precursors, e.g., the fading of memory traces of the behaviour [3,26]. For the present analysis, it is not critical if a shorter delay gradient in ADHD is caused by reduced WM capacity or if both are expressions of underlying dysfunctioning dopamine systems. Further, WM capacity is not necessarily a fixed entity and may also be modified by learning: a recent study reports that WM was improved in ADHD children by computerized training [27]. The present findings indicate that the delayed learning of a new task is related to reduced predictability in consecutive responding of ADHD individuals and not, for instance, to increased activity in general [14] or to increased perseveration, which would be the opposite of low predictability.

There might be alternative motivational explanations (than a shorter delay gradient) for the behaviour observed in the ADHD group. Rather than a reduced effect of positive reinforcement (i.e., the delivery of a reinforcer), the behaviour may be a result of reduced negative control (i.e., reduced compliance). However, non-compliance would have involved refusal to complete the task, which none of the children did, and most of them even reported that the task was fun. In addition, the instruction was non-directive, excluding the possibility of non-compliance to any instructed response pattern. A group of children aged 9–12 yr also participated in the original study, but the results of the comparison group indicated that their responding was mainly compliant and not controlled by reinforcers, as shown by lack of schedule control [14]. The behaviour of the younger children that participated in the present analysis did show schedule control, but the response patterns were different.

The present study provides new insights into the recently started discussion of intra-individual ADHD-related variability (ARV; [28]). Castellanos et al [28] calls for systematic studies of moment-to-moment changes in ongoing behaviour and to integrate such studies into causal models. The present study shows that ARV is found in other domains than reaction times, that it may be influenced by reinforcement contingencies, and that it might be explained by a short and steep delay gradient. Although the present study is purely behavioural, it might provide a framework within which to analyze several parallel processes, including cardiovascular and neurophysiologic, as suggested by Castellanos et al [28].

Some limitations of the present study could be improved in future studies. First, the findings need to be replicated in a larger sample of children. New samples, including girls and children of other ages, need to be analyzed in order to verify if this behavioural style is general to ADHD, whether it may be identified at an earlier age, and whether it continues to constitute an important factor in the behaviour of older children. Children with other psychopathologies should be added in order to establish the specificity of this learning style. Second, the present task may be criticized for its lack of ecological validity. On the other hand, the present task may have been optimal for studying ordering and sequencing of responses, as no earlier experience or learning history would interfere. In the future, a wide range of serial or sequential tasks should be investigated because the dynamic developmental theory of ADHD predicts that the presently-observed learning style should be found in the behaviour of people with ADHD across tasks and activities. Finally, various serial tasks could be conducted during brain imaging in order to investigate the brain areas involved.

## Conclusion

The present findings provide support for the dynamic developmental theory of ADHD predicting that a short and steep delay-of-reinforcement gradient will result in fewer responses in a predictable sequence than when the delay gradient is normal [2]. The hypothesized difference in timing patterns did not appear in the present study, as none of the groups showed any predictability in timing patterns.

The present study represents a new approach to analyzing the micro-dynamics of consecutive responses in a moment-to-moment manner. A majority of the responses were emitted with IRT < 1s. The study of behavioural processes and their environmental correlates may thus approach the time-scale of brain processes and we may get closer to directly measure brain-behavioural interactions. In this perspective, concepts like working memory, inhibition, and even timing problems may be too wide concepts to identify the underlying learning style, because a learning style characterized by inefficient chunking or development of entire behavioural sequences may be manifest in different domains in different individuals. One might speculate that sequencing of motor action and sequencing of speech and thought may be of the same functional origin (cf., [29]). Thus, sequencing deficits in ADHD may cause problems with rule-governed behaviour and self-control typical in ADHD behaviour.

## Competing interests

The author(s) declare that they have no competing interests.

## Authors' contributions

HA participated in the development of the study design, development of the reinforcement task used, carried out the data collection, prepared the data, performed the statistical analyses, and wrote the manuscript. TS participated in the development of the study design, development of the reinforcement task, wrote the programs for statistical analyses, participated in data analyses, read the manuscript and approved the final draft.
